# Prevalence of Fusion Between Adjacent Thoracic Spinous Processes in Adult Cadavers

**DOI:** 10.7759/cureus.103198

**Published:** 2026-02-08

**Authors:** Priyanka N Sharma, Kinjal Jethva, Hetal Vaishnani, Meghana Joshi, Priyanka Gohil, Manoj M Kulkarni, Achleshwar R Gandotra

**Affiliations:** 1 Anatomy, Smt. B. K. Shah Medical Institute and Research Centre, Sumandeep Vidyapeeth (Deemed to be University), Vadodara, IND

**Keywords:** scoliosis, spinous process fusion, thoracic spine, thoracic vertebrae, vertebral synostosis, wrong-level spine surgery

## Abstract

Introduction: Thoracic vertebrae play a crucial role in spinal stability and the protection of the spinal cord. Thoracic spinous process fusion, whether congenital or acquired, can affect spinal biomechanics and complicate surgical procedures. Thoracic spinous process fusion is an anatomical variation with potential clinical significance; however, its prevalence and distribution remain underreported. This study aimed to determine the prevalence and vertebral-level distribution of thoracic spinous process fusion in adult cadavers.

Methods: A cross-sectional observational cadaveric study was conducted on 30 formalin-fixed adult cadavers (15 males, 15 females) aged 60-95 years. After dissection and removal of soft tissues, the thoracic spinous processes from T1 to T12 were examined for osseous fusion between the adjacent vertebrae. Fusion was defined as complete bony continuity without interspinous gaps or fibrous tissues. The presence of fusion and vertebral levels was documented. Descriptive statistics and Fisher’s exact tests were used for the analyses.

Results: Thoracic spinous process fusion was observed in five of 30 thoracic spine specimens (360 thoracic vertebrae) (16.7%), with two men (6.7%) and three women (10.0%) affected. Fusion occurred at the T3-T4, T6-T7, T9-T12, and T11-T12 levels, with T11-T12 showing the highest frequency (6.7%). No statistically significant sex differences were found (p=1). All fused spinous processes demonstrated complete bony continuity without the presence of fibrous tissue.

Conclusion: Thoracic spinous process fusion is a notable anatomical variation, with a prevalence of 16.7% in the adult cadaveric sample. Awareness of its prevalence and localization can assist surgeons in preoperative planning and intraoperative level identification, thereby reducing surgical errors and improving patient outcomes.

## Introduction

The thoracic vertebrae (T1-T12) are intermediate in size between the cervical and lumbar vertebrae and exhibit characteristic anatomical features associated with rib articulation. They play a pivotal role in maintaining spinal stability and protecting the spinal cord. [1-4] Each thoracic vertebra consists of a cylindrical vertebral body and a posterior vertebral arch formed by pedicles and laminae, which unite posteriorly to form a long, inferiorly directed spinous process. The typical thoracic vertebral body is heart-shaped and bears superior and inferior costal facets on its lateral aspects for articulation with the rib cage. The transverse processes also possess costal facets, except in T11 and T12, where the ribs are absent. The laminae and spinous processes overlap obliquely in a roof tile arrangement, providing posterior protection to the spinal cord. The vertebral foramina at the thoracic level are relatively smaller, corresponding to the reduced diameter of the spinal cord. In the mid-thoracic region, the spinous processes are long and steeply inclined, with the tips lying one to one-and-a-half vertebral levels below the corresponding transverse processes, whereas in the upper and lower thoracic regions, they are less obliquely oriented. The intervertebral foramina, located between adjacent pedicles, transmit the segmental thoracic spinal nerves and are positioned predominantly behind the vertebral bodies, making nerve root compression less common in the thoracic spine than in the lumbar region [1-3].

The vertebral column represents a fundamental expression of body segmentation, with vertebrae and intervertebral discs developing through a precisely regulated embryological process [1]. During the fourth week of intrauterine life, the sclerotome portion of the somites migrates around the notochord and neural tube and undergoes resegmentation, forming individual vertebrae. Disruptions in this process may result in vertebral anomalies, including partial or complete fusion of adjacent vertebrae, commonly referred to as vertebral synostosis, block vertebrae, or spinal fusion [5].

Vertebral fusion can be either congenital or acquired. Congenital fusion results from the failure of normal segmentation and may occur as an isolated anomaly or as part of syndromic conditions such as Klippel-Feil syndrome, or may cause spine deformations such as scoliosis. Acquired fusion of vertebrae is due to diseases such as tuberculosis, juvenile rheumatoid arthritis, and trauma [3, 5]. The presence of fused vertebrae results in biomechanical stress in the adjoining segments, leading to premature degenerative changes in the adjoining motion segments. The wasp waist sign, characterized by an anterior concave indentation where the interspace between fused vertebrae is absent or reduced on radiographs, is associated with complete vertebral fusion in Klippel-Feil syndrome. Fusion of vertebrae can occur in a sequence of common occurrences in the cervical, lumbar, and thoracic regions. Surgical fusion of the vertebrae is known as spondylodesis or spondylosyndesis. It can also be congenital or acquired. The cervical spine is invariably an anomalous constitution in clinical cases of Willet-Sprengel shoulder, brevicollis, kyphosis, congenital deafness, renal agenesis, and cardiovascular abnormalities. Vertebral synostosis is associated with radiculopathy and myelopathy. Vertebral synostosis is associated with radiculopathy and myelopathy and may be seen in association with various syndromic conditions, including congenital segmentation disorders and musculoskeletal anomalies [3, 6].

Spinous processes serve as important attachment sites for paraspinal muscles and ligaments and act as palpable anatomical landmarks during clinical examinations and spinal procedures [1]. Fusion of the spinous processes, particularly in the thoracic region, has received comparatively less attention than vertebral body fusion. Given the role of the thoracic vertebrae in maintaining thoracic cage integrity and spinal alignment, such anomalies may contribute to abnormal spinal curvatures and functional compromise [5].

Despite their anatomical and clinical relevance, data on the prevalence and vertebral level distribution of fusion between adjacent thoracic spinous processes are limited. Therefore, the present study aimed to determine the prevalence and distribution of thoracic spinous process fusion in adult cadavers.

## Materials and methods

This observational cadaveric study was conducted at the Department of Anatomy of Smt. B. K. Shah Medical Institute and Research Centre, a teaching medical institute in Vadodara, Gujarat, India. Ethical approval for the present study was obtained from the Institutional Ethics Committee (Sumandeep Vidyapeeth Institutional Ethics Committee, Reference Letter No.- SVIEC/OW/MEDI/PHD/18005). 

The study included 30 formalin-fixed adult human cadavers (western Indian population), comprising 15 males and 15 females, aged between 60 and 95 years (mean age: 77.5 ± 10.1 years). The sample size was determined by cadaver availability during the study period. Cadavers with external deformities, evidence of trauma, pathological lesions, or prior surgical interventions involving the vertebral column were excluded from the study. Each cadaver was placed in the prone position on a flat surface for dissection. A midline posterior incision was made in the thoracic region. The superficial and deep muscles of the back were carefully dissected and removed to expose the thoracic vertebral column from T1 to T12. The spinous processes of individual thoracic vertebrae were meticulously cleaned and examined for fusion between adjacent thoracic spinous processes.

Thoracic spinous process fusion was defined as complete osseous continuity between adjacent spinous processes, characterized by the absence of an interspinous gap, fibrous tissue, or a visible fusion line, with continuous bony contours. In cases where differentiation between osseous and fibrous continuity was uncertain, gentle scraping was performed to confirm the type of fusion. The vertebral level fusion was assessed between adjacent spinous processes along the midline. High-resolution photographs were obtained for documentation and analyses by a single experienced observer using standardized measurement techniques. This approach minimized interobserver variability. Data collection and analysis were performed using IBM SPSS Statistics, version 23.0 (IBM Corp., Armonk, NY, USA). The statistical analysis was primarily descriptive. Categorical variables are expressed as frequencies and percentages. The occurrence of spinous process fusion was compared between male and female specimens using Fisher’s exact test, considering the small sample size and low expected cell count. Statistical significance was set at p < 0.05. Owing to the low frequency of fusion at the individual vertebral levels, only descriptive statistics were applied for level-wise analysis.

## Results

A total of 30 thoracic spine specimens (360 thoracic vertebrae) were analyzed, consisting of 15 male and 15 female specimens. Fusion of the spinous process was observed in five specimens (16.7%).

Among the male specimens, fusion was identified in two specimens (6.7%). Fusion was observed at the T9-T12 level in one specimen (3.3%) and at the T11-T12 level in another (3.3%). No fusion was observed in the male specimens at the T3-T4 or T6-T7 levels.

**Figure 1:**
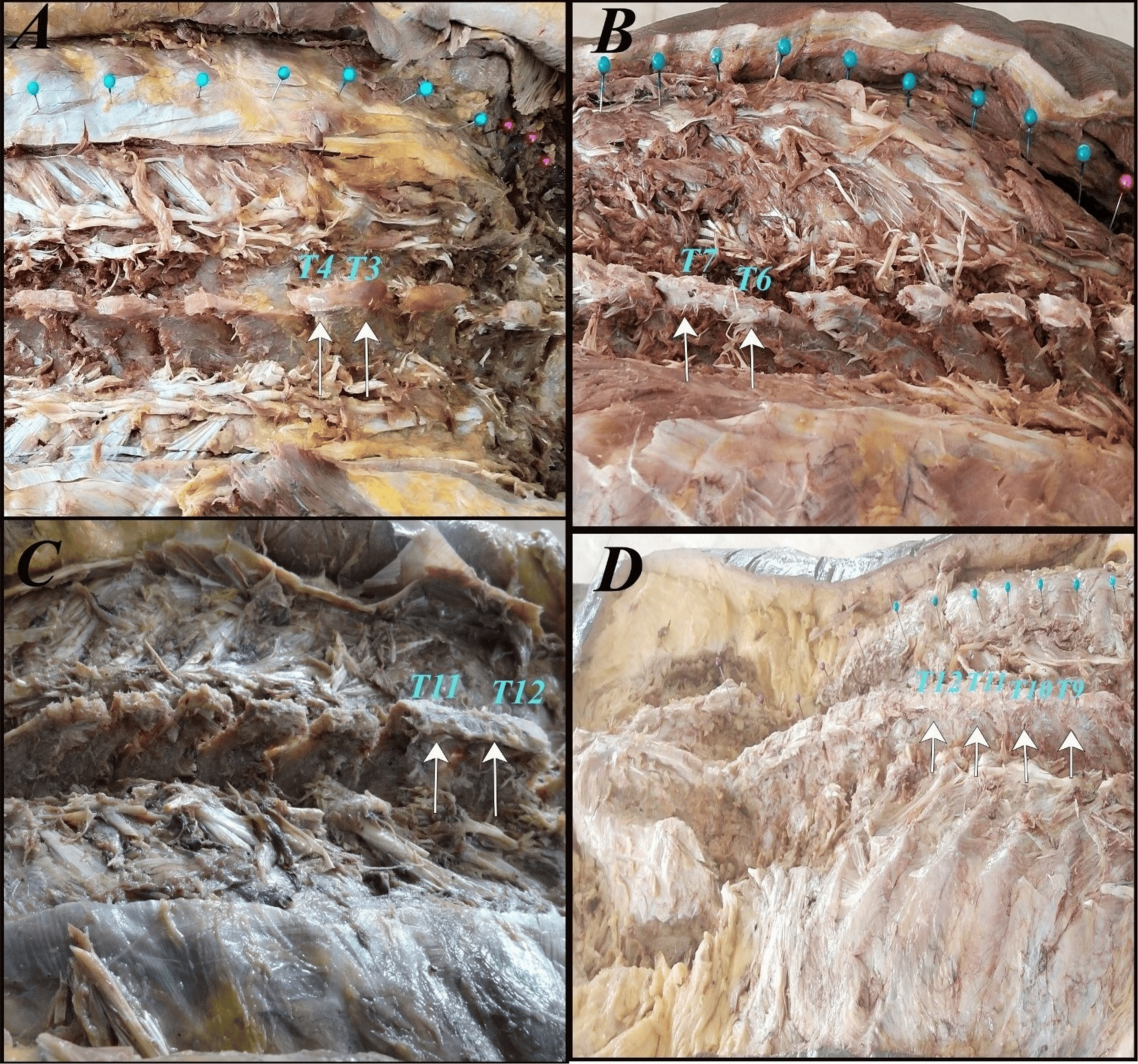
Thoracic spinous process fusion (white arrow) at A) T3-T4, B) T6-T7, C) T11-T12, and D) T9-T12.

Among the female specimens, fusion was present in three specimens (10.0%). Fusion at the T3-T4 and T6-T7 levels was observed in one specimen each (3.3% per level). Fusion at the T11-T12 level was also observed in one specimen (3.3%). No fusion was identified in the female specimens at the T9-T12 levels (Figure 1).

Overall, fusion at the T3-T4, T6-T7, and T9-T12 levels accounted for 3.3% of the total specimens examined, whereas the T11-T12 level showed the highest frequency, with fusion observed in two specimens (6.7%) (Table 1). All specimens demonstrated complete osseous continuity without visible fusion lines, which was consistent with bony fusion. The transverse processes of the adjacent vertebrae did not exhibit fusion. On comparison between sexes, the difference in the occurrence of thoracic spinous process fusion was not statistically significant (Fisher’s exact test, p = 1.00).

**Table 1:** Frequency of thoracic spinous process fusion

The fusion of the spinous process	Sample size (Total=30, Male=15 and Female=15)			
	Male specimen showing fusion (%)	Female specimen showing fusion (%)	Total number of specimens showing fusion (%)	Fisher’s exact test
T3-T4	-	1 (3.3)	1 (3.3)	p = 1.00
T6-T7	-	1 (3.3)	1 (3.3)	
T9 – T12	1 (3.3)	-	1 (3.3)	
T11-T12	1 (3.3)	1 (3.3)	2 (6.7)	

## Discussion

Thoracic spinous process fusion, although less commonly reported than vertebral body fusion, is of considerable clinical significance in procedures such as thoracic decompression, laminectomy, pedicle screw fixation, spinal fusion, and deformity correction surgeries. Awareness of this anatomical variation is essential to optimize surgical planning, maintain spinal biomechanics, and ensure accurate intraoperative level localization, thereby reducing the risk of wrong-level spine. The present cadaveric study assessed the prevalence and vertebral distribution of thoracic spinous process fusion and documented an overall incidence of 16.7%. This prevalence is notably higher than that reported in earlier studies by Sharma et al. [7], Deepa et al. [8], Kulkarni et al. [6], Nazeem et al. [5], and Sar et al. [9], who reported fusion rates of 4.16%, 4%, 0.37%, 0.18%, and 0.11%, respectively (Table 2). The higher incidence observed in the present study may be attributed to the older age group examined (60-95 years) and the systematic cadaveric dissection method, which allows for direct visual and tactile assessment of the osseous continuity. Fusion was identified at multiple thoracic levels, with variations observed between male and female specimens; however, these differences were not statistically significant. These findings add meaningful baseline anatomical data to the limited body of literature on thoracic spinous process fusion, an anatomical variation with important clinical implications.

**Table 2:** Comparison of thoracic spinous process fusion with other studies

Authors	Sample size	Fusion (%)
Kulkarni V et al. (2012) [6]	270 dry vertebrae	0.37
Sharma M et al. (2013) [7]	48 dry vertebral columns	4.16
Deepa S et al. (2014) [8]	50 dry vertebral columns	4.0
Nazeem et al. (2014) [5]	550 dry vertebrae	0.18
Sar M et al. (2017) [9]	856 dry vertebrae	0.11
Present study	30 cervical spine specimens	16.7

From an embryological perspective, the vertebral column develops from paired somites, each comprising a dermatome, myotome, and sclerotome. During the fifth week of development, sclerotomal cells migrate to form the vertebral body, neural arch, and costal elements of the ribs. Disruption of this segmentation process results in non-segmentation of the sclerotome, leading to congenital vertebral fusion or block vertebrae [3, 5-8]. Radiologically, congenital vertebral fusion is characterized by the absence of an intervertebral disc or its replacement by a radiopaque line, the presence of a wasp-waist appearance, smooth intervertebral foramina, and a single spinous process spanning two vertebral bodies, with preservation of vertebral body height [3, 10].

Vertebral fusion alters the normal spinal anatomy and biomechanics and may predispose individuals to abnormal spinal curvatures, early degenerative changes, and adjacent segment disease later in life. Fusion between the thoracic and lumbar vertebrae has been associated with low back pain, disc prolapse, and postural imbalance, particularly in older individuals [3, 5, 10-14]. Early identification of such anomalies is clinically valuable, as it aids in distinguishing congenital variations from acquired pathologies related to trauma, aging, or degenerative progression, and allows for timely lifestyle modifications and clinical interventions [3, 13, 14].

Congenital block vertebrae may coexist with various systemic anomalies, including scoliosis, Sprengel’s deformity, hemivertebrae, platybasia, basilar impression, spina bifida, clubfoot, renal and rib anomalies, cleft palate, respiratory compromise, hearing impairment, and congenital cardiac defects [5, 6, 8, 11, 13, 15, 16]. Vertebral fusion is also a recognized component of several segmentation syndromes, including those associated with laryngeal malformations [3, 5, 6, 10, 14, 17-20].

The thoracic spine presents inherent challenges for accurate intraoperative level localization because of its overlapping spinous processes, rib articulations, and reduced mobility. Thoracic spinous process fusion further complicates this process, increasing the risk of wrong-level spine surgery, a well-recognized patient safety concern with significant medicolegal consequences. Spine surgery is particularly vulnerable to wrong-level errors because surgeons must accurately identify both the correct side and vertebral level [10, 17, 21]. Previous reports have indicated that nearly 50% of spine surgeons have performed at least one wrong-level procedure during their careers [22-28]. Large-scale analyses have shown that although wrong-level surgeries occur most frequently in the lumbar spine, approximately 8% involve the thoracic region, where anatomical complexity is greatest [17, 29, 30].

Accurate thoracic level localization is further challenged by factors such as obesity, osteoporosis, scapular shadowing, anatomical variation in rib-bearing vertebrae, and distance from fixed anatomical landmarks [10, 17, 23, 24, 26-28, 31]. Failure to anticipate these difficulties may result in prolonged operative times, wrong-site surgeries, or suboptimal clinical outcomes. Therefore, meticulous preoperative imaging, thorough anatomical understanding, and intraoperative verification techniques, including fluoroscopy, fiducial markers, and reliance on consistent anatomical landmarks, are essential for minimizing surgical errors and improving patient safety.

Embryologically, vertebral fusion arises from segmentation defects during early development, whereas acquired fusion may result from trauma, infection, or inflammatory conditions [3, 5]. In the present study, the observed thoracic spinous process fusion likely represents a combination of congenital factors and age-related degenerative changes like sclerotome segmentation and resegmentation defects during early embryogenesis, given the advanced age of the examined cadaveric population.

Limitations

Histological confirmation was not undertaken, and the cadaveric nature of the study precluded definitive differentiation between congenital and acquired fusion. Additionally, the lack of radiological correlation limited clinical interpretation for surgical planning, while the advanced age of the specimens may have contributed to the findings due to degenerative or systemic ankylosing conditions.

## Conclusions

Thoracic vertebral fusion is a notable variation that may be congenital or acquired. In this study, it was typically found at the T9-L5 and T11-T12 levels in males and at the T3-T4, T6-T7, and T11-S1 levels in females. These findings enhance our understanding of the morphology, embryological basis, and clinical implications of thoracic spinous process fusion. Awareness of its prevalence and vertebral-level distribution is important for thoracic spine surgeries, including decompression, laminectomy, pedicle screw fixation, spinal fusion, and deformity correction, to facilitate accurate level localization and reduce the risk of wrong-level spine surgery.
